# Downregulation of *MALAT1* is a hallmark of tissue and peripheral proliferative T cells in COVID-19

**DOI:** 10.1093/cei/uxad034

**Published:** 2023-03-03

**Authors:** Shoumit Dey, Helen Ashwin, Luke Milross, Bethany Hunter, Joaquim Majo, Andrew J Filby, Andrew J Fisher, Paul M Kaye, Dimitris Lagos

**Affiliations:** Hull York Medical School and York Biomedical Research Institute, University of York, York, UK; Hull York Medical School and York Biomedical Research Institute, University of York, York, UK; Newcastle University Translational and Clinical Research Unit, Faculty of Medical Sciences, Newcastle University, Newcastle upon Tyne, UK; Flow Cytometry Core Facility and Innovation, Methodology and Application Research Theme, Newcastle University Biosciences Institute, Newcastle University, Newcastle upon Tyne, UK; Department of Cellular Pathology, Newcastle Upon Tyne Hospitals NHS Foundation Trust, Newcastle upon Tyne, UK; Flow Cytometry Core Facility and Innovation, Methodology and Application Research Theme, Newcastle University Biosciences Institute, Newcastle University, Newcastle upon Tyne, UK; Newcastle University Translational and Clinical Research Unit, Faculty of Medical Sciences, Newcastle University, Newcastle upon Tyne, UK; Institute of Transplantation, Newcastle upon Tyne Hospitals NHS Foundation Trust Newcastle upon Tyne, UK; Hull York Medical School and York Biomedical Research Institute, University of York, York, UK; Hull York Medical School and York Biomedical Research Institute, University of York, York, UK

**Keywords:** T cell, proliferation, MALAT1, COVID-19, lncRNA

## Abstract

T cells play key protective but also pathogenic roles in COVID-19. We studied the expression of long non-coding RNAs (lncRNAs) in COVID-19 T-cell transcriptomes by integrating previously published single-cell RNA sequencing datasets. The long intergenic non-coding RNA *MALAT1* was the most highly transcribed lncRNA in T cells, with Th1 cells demonstrating the lowest and CD8+ resident memory cells the highest *MALAT1* expression, amongst CD4+ and CD8+ T-cells populations, respectively. We then identified gene signatures that covaried with *MALAT1* in single T cells. A significantly higher number of transcripts correlated negatively with *MALAT1* than those that correlated. Enriched functional annotations of the *MALAT1*- anti-correlating gene signature included processes associated with T-cell activation such as cell division, oxidative phosphorylation, and response to cytokine. The *MALAT1* anti-correlating gene signature shared by both CD4+ and CD8+ T-cells marked dividing T cells in both the lung and blood of COVID-19 patients. Focussing on the tissue, we used an independent patient cohort of post-mortem COVID-19 lung samples and demonstrated that *MALAT1* suppression was indeed a marker of MKI67+ proliferating CD8+ T cells. Our results reveal *MALAT1* suppression and its associated gene signature are a hallmark of human proliferating T cells.

## Teaser

T cells play important roles in the body’s response to COVID-19. We found gene signatures anti-correlating to MALAT1 expression as a marker of proliferating CD4+ Th1 and CD8+ exhausted sub-populations.

## Graphical Abstract

**Figure:**
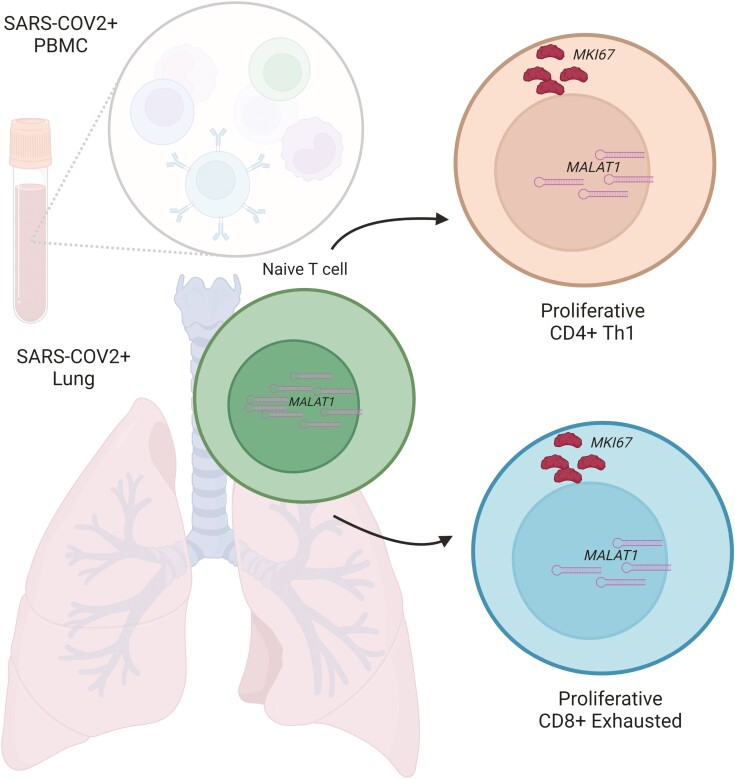

## Introduction

T-cell plasticity and balance are crucial for protection and pathology [1, 2]. Such opposing roles have been appreciated in various provocations of the immune system and more recently in the progression of COVID-19 [3, 4]. While antigen-specific T cells may confer protection against SARS-CoV-2 virus [5–7], lymphopenia is associated with severe COVID-19 [8–10] and exhausted, senescent T cells and those expressing MKI67 [11, 12], a key proliferation marker, contribute to pathology [3, 13–15]. In CD8+ T cells, a strongly proliferative phenotype correlates with contraction and disappearance of clones in acute COVID-19 pathology [16].

Long non-coding RNAs (lncRNAs) are regulatory non-coding RNAs, longer than 200nt. In most cases, lncRNAs show low-medium expression with poor conservation across species often acting as scaffolds for recruitment, sequesters for chromatin-modifiers, or RNA binding proteins to specific genomic sites [17, 18]. LncRNAs may be *cis-* or *trans-*acting wherein the former influences transcription by affecting the loci near their transcription site (enhancer-like) while the latter transcripts leave the transcription site to affect gene expression (mRNA-like) via transcriptional or post-transcriptional mechanisms [19].

LncRNAs play essential roles in adaptive immunity, particularly in lymphocyte activation, signaling and effector functions [20]. For example, lncRNAs such as *lncHSC-2* commit HSCs to lymphoid specification as B or T cells [21]. T-cell development is regulated by Notch1 signaling [22] whose expression is in turn regulated by the lncRNA *NALT1* [23]. As T cells mature, their activation is triggered by T-cell receptors (TCRs) upon MHC-mediated antigen presentation that is further modulated by co-stimulatory or co-inhibitory ligands. This activation leads to a switch to glycolysis [24] which is in turn is influenced by the lncRNA *PVT1* [25, 26]. Indeed, sets of lncRNAs specifically regulate lineage-specific gene expression in activated T cells [27] such as Th1 [28], Th2 [29], Th17, [30] and Treg [31] programs.

Profiling lncRNA expression in immune cells during the response to infection can provide insights into key transcriptional and post-transcriptional mechanisms operating in health and disease. Of note, even though the transcriptomes of tissue and peripheral T cells during responses to infection, and more specifically SARS-CoV-2 have been extensively studied [32], the study of T-cell lncRNA profiles has been limited [33, 34].

We explored T-cell lncRNA profiles from three publicly available datasets from individuals with COVID-19 identifying several lncRNAs that are detectable in lung T cells during infection. We particularly focused on *MALAT1*, a long intergenic non-coding RNA (lincRNA, a sub-class of lncRNAs) remarkably conserved in vertebrates [35]. LincRNAs such as MALAT1 do not overlap with protein-coding genes and can have various regulatory effects on gene expression [36]. Localized in nuclear speckles [37, *MALAT1* is known to work in a variety of ways, such as through binding splicing factors [37], controlling the function of proteins involved in transcription [38], miRNA sequestration [39], and associating with proteins [40]. *MALAT1* has been associated with positively regulating cell cycle progression in cancer tissues [41] a loss of which impairs cell proliferation [23]

*MALAT1* has been shown to regulate T-cell function, predominantly in animal models of infection or immunopathology [42–45]. In a previous study, in CD4+ T cells, we reported that MALAT1 downregulation is a hallmark of naïve CD4+ T-cell activation and that *MALAT1*−/− CD4+ T cells express lower levels of IL-10, an anti-inflammatory cytokine resulting in enhanced inflammation or immunity in experimental models of leishmaniasis and malaria [46].

Here, we examined COVID-19 single-cell RNA sequencing (scRNA seq) datasets from bronchoalveolar lavage (BAL) [47, 48], explant/post-mortem lung cells [49], and peripheral blood [50] and discovered that *MALAT1* was negatively correlated with cell cycle progression and proliferation in CD4+ and CD8+ T cells of severe COVID-19 patients. Performing RNAscope on COVID-19 post-mortem lung tissue from individuals who died of COVID-19, we confirmed that MKI67-expressing CD8+ T cells had lower levels of *MALAT1* mRNA *in situ*. Overall, our findings reveal that MALAT1 expression in T cells from COVID-19 patients is linked to a specific gene signature and that low *MALAT1* expression is a hallmark of proliferative T cells.

## Results

### MALAT1 is differentially expressed in CD4+ and CD8+ subpopulations

**Figure 1.**
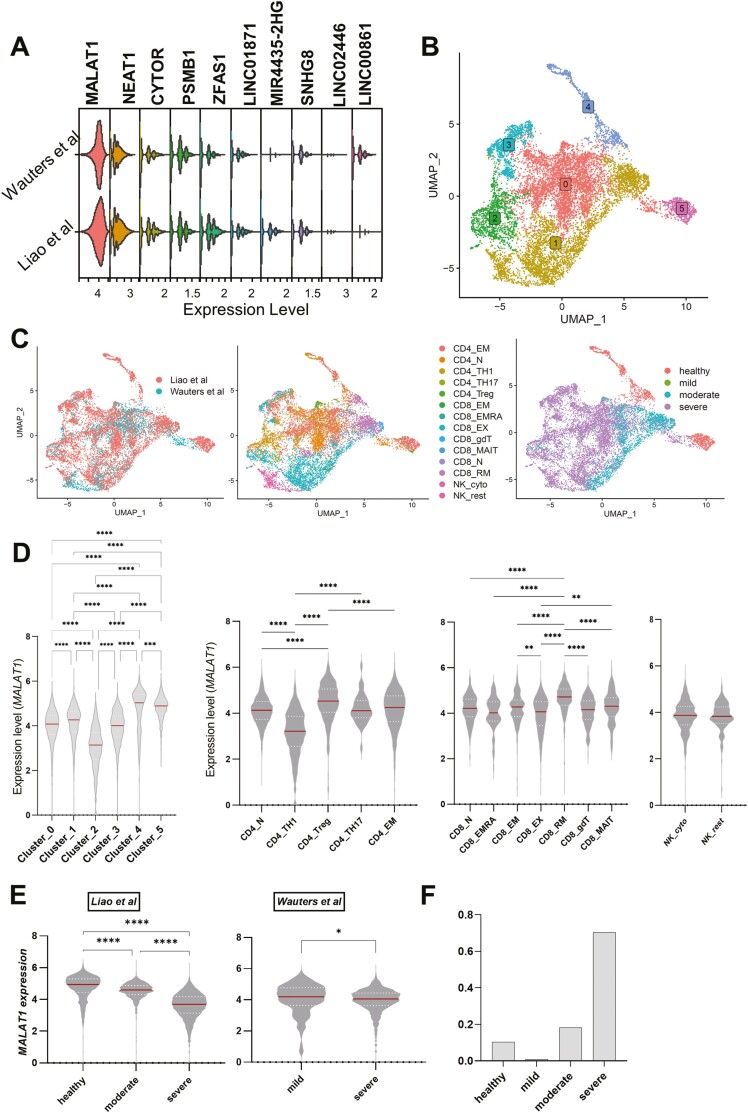
*MALAT1* is differentially expressed in T cell subsets: (**A**) Stacked violin plots showing the top 10 highly expressed LncRNA in T cells in bronchoalveolar lavage fluid studies. (**B**) UMAP plots depicting single cells colored by their cluster identity as indicated by colored boxed labels. (**C**) UMAP plots depicting single cells colored and labeled by their respective study (Liao *et al*. (2020) and Wauters Mol *et al*. (2021)). Same as left but colored by imputed cell sub-population type and disease severity (healthy = 1225 (Liao *et al*. only), mild = 111, moderate = 2135, severe = 8275 T cells), respectively. (**D**) Violin plots showing normalized counts of *MALAT1* expression across cluster identities of T cells and imputed cell sub-populations. Kruskal–Wallis multiple comparison *P*-values are indicated by asterisks (****P < 0.0001, ***P = 0.0001–0.005,**P = 0.005–0.001,*P = 0.01–0.05). For cell sub-populations, only a subset of comparisons is shown. (**E**) Violin plots showing normalized counts of *MALAT1* expression across study and disease severity. Kruskal–Wallis multiple comparisons with p-value asterisks as defined in (**D)**. (**F**) Barplot showing proportion of total CD4+/CD8 + T cells represented by each severity group pooled for both datasets.

We integrated T-cell BAL scRNAseq datasets [47, 48] to look at highly expressed lncRNAs in T cells from healthy volunteers and individuals with COVID-19 (Methods; Fig. 1A). We found *MALAT1* to be the highest expressed lncRNA with similar distribution in both datasets which is ubiquitously found across all T cells (Fig. 1A). We then normalized and integrated the two datasets (see Methods) and clustered them at a low resolution to infer coarse-grained T-cell heterogeneity (Fig. 1B; Supplementary Fig. S1). Cells visualized on UMAP showed both the datasets to be similarly spread across UMAP space indicating similar composition (Fig. 1C left). We then used cell type metadata [47,48] to obtain a finer-grained T-cell phenotyping (Fig. 1C middle). We found that there were marked differences between T cells based on disease severity (Fig. 1C right).

Importantly, we found that *MALAT1* is differentially expressed within unbiased clusters, especially cluster 2 (Fig. 1D left) and within imputed T-cell subpopulations. We found that Th1 cells (CD4_TH1, inflammation-associated TNF/IFNγ expressing effector cells) demonstrate lower *MALAT1* levels with respect to naïve CD4+ T cells (CD4_N, immature cells with no exposure to cognate antigen), confirming previous findings in mouse Th cells [46]. CD4_Treg (regulatory T cells) showed the highest *MALAT1* levels. We also observed differences in *MALAT1* expression within CD8+ T cells, with CD8_RM subset showing the highest *MALAT1* expression compared to all other subsets. The difference in *MALAT1* expression between CD8_RM (tissue-resident memory CD8) and CD8_EM/CD8_EMRA (memory cells/recently activated memory cells in periphery) may mark how a memory T cell is poised toward tissue homing [51]. While exhausted CD8+ T cells (CD8_EX, activated cells with exhausted effector function) had a lower median value of MALAT1 than naïve CD8+ cells (CD8_N), this was not significant. However, compared to CD8_EM, CD8_EX had lower MALAT1 levels. It is notable that the lower quartile of CD8_EX cells was the lowest among all CD8 subsets (Fig. 1D right). In our data integration (see Methods), we retained cell cycle genes, as *MALAT1* has been previously linked to the cell cycle [23, 41]. In doing so, and as suggested [47, 48], we found cluster 2 (Fig. 1B) to be a mix of CD4_TH1 and CD8_EX T cells (Fig. 1C, middle panel). Interestingly, *MALAT1* expression was reduced in T cells from BAL from severe patients in both the datasets (Fig. 1E), although we note that this may be biased due to the low proportion of cells from non-severe patients (Fig. 1F). Interestingly, among the top 10 highly expressed lncRNAs (Fig. 1A; Supplementary Fig. S2) only *MALAT1* seemed to be down-regulated in severe cases with respect to both healthy, mild/moderate cells (Fig. 1E versus Supplementary Fig. S2).

### MALAT1(anti-)correlated gene lists identify CD8 + T_EX_ CD4+ T_TH1_ Cells

**Figure 2.**
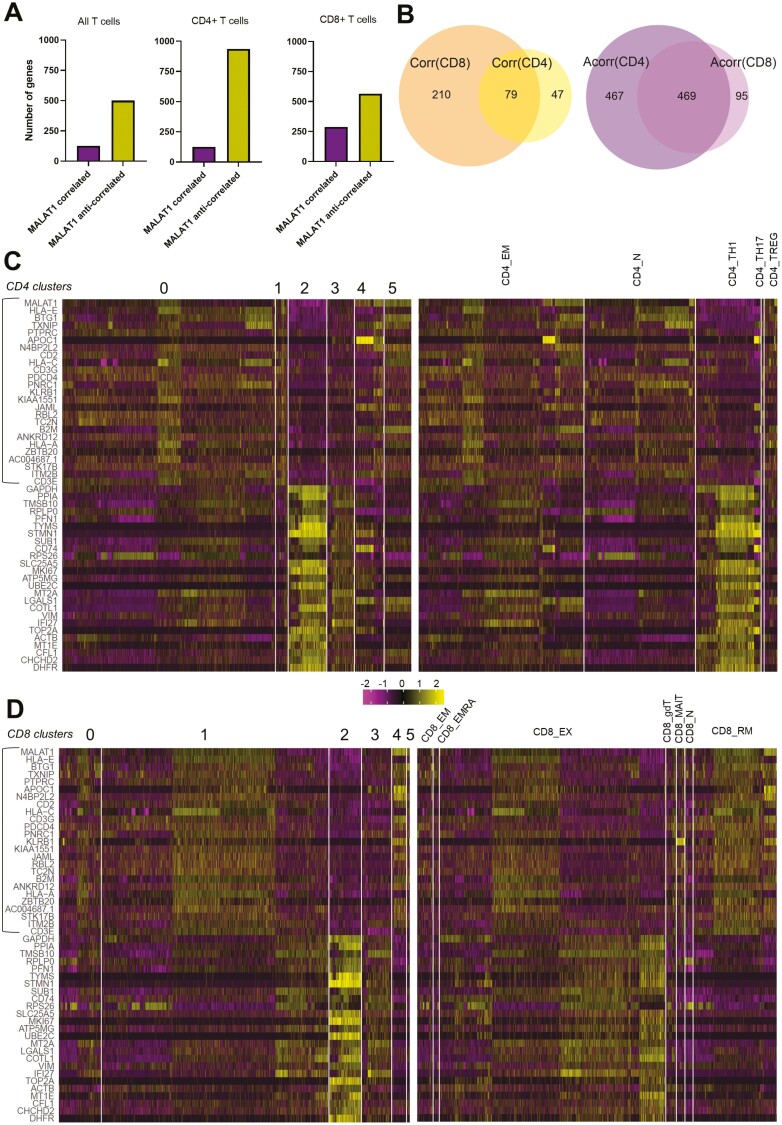
BAL T-cell sub-populations differentially express MALAT1-correlated genes. (**A**) Bar plots showing the number of genes that correlate or anti-correlate with MALAT1 for all T cells, CD4+ and CD8+ T cells. (**B**) Venn diagrams depicting the intersection of gene lists that correlated [Corr(CD8), Corr(CD4)] and that for those that anti-correlated [Acorr(CD8), Acorr(CD4)] with MALAT1 expression in CD4+ cells and CD8+  cells. (**C**) Heatmap of top 25 MALAT1 correlating genes (highlighted in the rectangle) and top 25 anti-correlating genes in CD4 + T cells grouped by their cluster identities and by sub-population. Heatmap legend indicates expression values scaled to a mean of zero. (**D**) Same as C but for CD8+ T cells.

To understand the effect of variability in *MALAT1* expression (Fig. 1D) across coarse- and fine-grained T-cell heterogeneity we looked at how *MALAT1* gene expression correlated against all other genes across all T cells, or only CD4+ T cells or CD8+ T cells, respectively. Keeping a significance score of *P* = 0.05 and the positive correlation value > 0.1 or negative correlation value < −0.1 as a cut-off, we found that ~80% of the genes that significantly co-vary with *MALAT1* are those anti-correlated to its expression (all T cells, Fig. 2A). This percentage is ~88% for CD4+ T cells and ~65% for CD8+ cells when correlations were calculated separately for CD4+ and CD8+ cells (Fig. 2A).

Upon analyzing the intersection of these gene lists we found that out of the genes that correlated positively with *MALAT1* for CD4+ and CD8+ cells, there were 79 genes that were common between the two T-cell types while there were over four times as many uniquely *MALAT1* correlated genes in CD8+ over CD4+ cells (210 versus 47; Fig. 2B). While CD4+ and CD8+ cells shared a high number of genes that were anti-correlated to *MALAT1*, the number of genes that were unique in anti-correlation lists were four times as many in CD4+ T cells than CD8+ T cells (Fig. 2B).

We next identified whether the top 25 *MALAT1*-correlated and top 25 *MALAT1*-anti-correlated genes (based on correlation value) in both CD4+ T cells (Fig. 2C) and CD8+ T cells (Fig. 2D) were differentially expressed in clusters identified previously (Fig. 1D left) or within imputed cell sub-populations (Fig. 1D center). When grouped by cluster identities, in CD4+ T cells, *MALAT1* anti-correlated genes are upregulated in cluster 2 (bottom 25 genes, Fig. 2C) while a strong *MALAT1* correlated signature is observed in clusters 0, 1, and 5 (top 25 genes, Fig. 2C left). Cells in cluster 2 (Fig. 2C left) may predominantly be CD4_TH1 cells (Fig. 2C right) while clusters 0, 1, and 5 (Fig. 2C left) may largely comprise either CD4_N (naïve) or effector memory (CD4_EM) CD4+ T cells (Fig. 2C right).

In a similar manner in CD8+ T cells, cluster 2 is characterized by genes that are *MALAT1* anti-correlated (bottom 25 genes, Fig. 2D). When grouped by T-cell subpopulations, CD8+ T_EX_-cells appeared to be enriched in the *MALAT1* anti-correlated signature (Fig. 2D). When grouped by cluster identities, *MALAT1* anti-correlated genes were expressed in cluster 2 of CD8+ T cells (Fig. 2D) as with CD4+ T cells (Fig. 2C). Interestingly, CD8+ T_EX_-population appears heterogeneous in terms of expression of MALAT1 anti-correlating and correlating genes (Fig. 2D) which may explain why MALAT1 expression is not significantly different between CD8 T_N_ and CD8 T_EX_ cells (Fig. 1D right). In addition, *MALAT1* correlated signature is enriched in the resident memory subset (CD8+ T_RM_) and effector memory (CD8+ T_EM_) sub-populations (Fig. 2D).

Importantly, *MALAT1* is anti-correlated with *MKI67,* a commonly used marker of T-cell proliferation which is really a graded marker of the same and also marks T cells that may have recently divided [52], in both CD8+ and CD4+ and its expression is increased in cluster 2 (Fig. 2C and D) potentially indicating the proliferative nature of cells in this cluster. Overall, these findings identified a core gene signature that anti-correlates with MALAT1 expression in T cells and indicated that these genes were highly expressed in proliferative CD4_TH1 and CD8_EX cells.

### MALAT1 anti-correlated genes include a core proliferation and cell-specific signature in T cells

**Figure 3.**
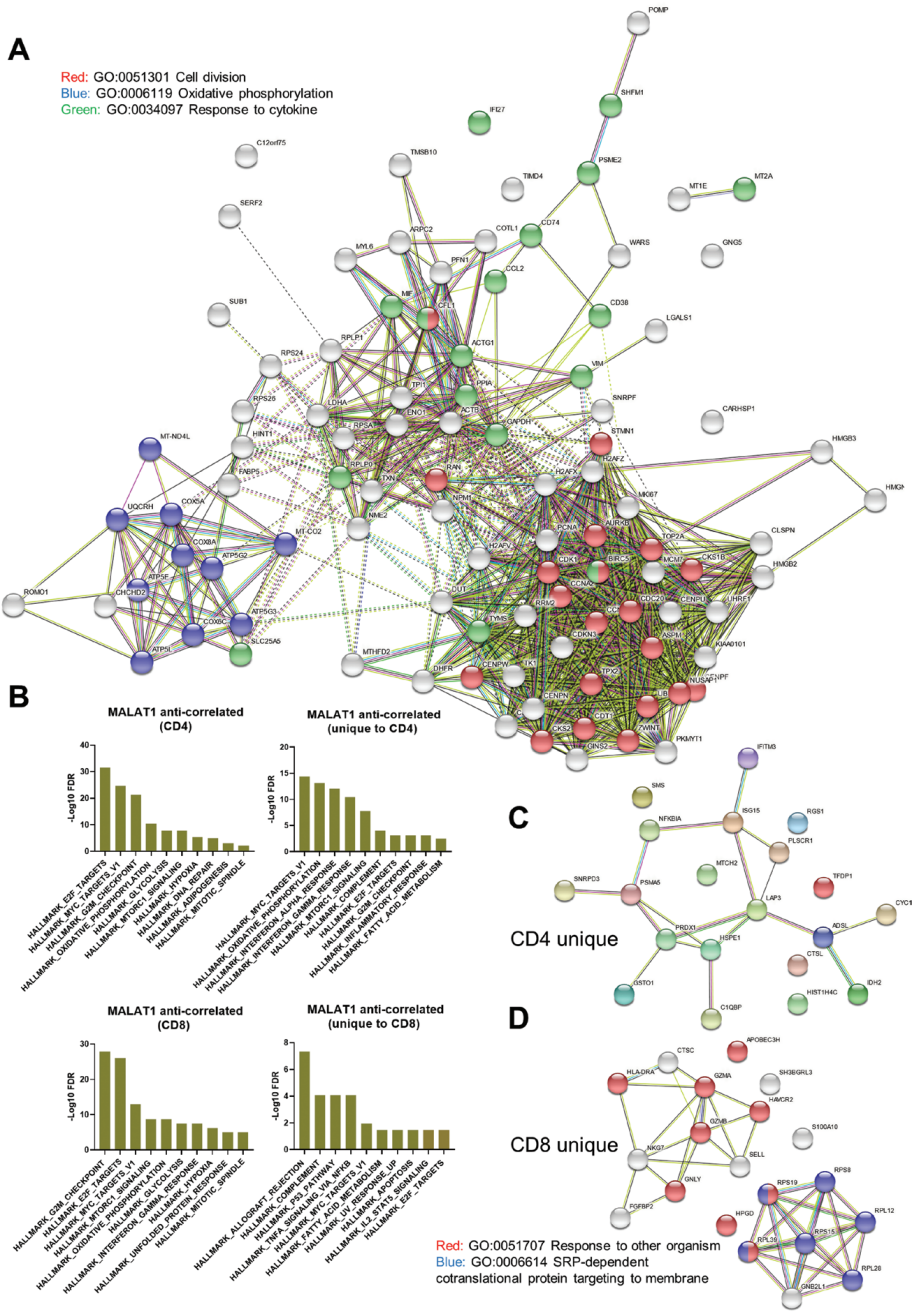
*MALAT1* anti-correlates to genes that are related to cell cycle progression in T cells. (**A**) Network representation of STRING interactions plotted for top 100 *MALAT1* anti-correlated genes and those that are common in CD4+ and CD8+ T cells with lines indicating interconnectedness in terms of co-expression or interaction. (**B**) Barplots showing negative log FDR values for HALLMARK GSEA enrichment for genes that anti-correlate with MALAT1 and those that are uniquely so in CD4+ and CD8+ T cells. The ‘*x*’ axis contains HALLMARK gene set names that were found to enriched. (**C**) Same as A but for top 20 unique genes for CD4+ T cells. (**D**) Network representation of STRING interactions for top 20 unique CD8+ T cells.

Next, we used STRING-DB to perform network analysis for the top 100 genes (corresponding to approximately the top 25th percentile of all correlation values) that anti-correlate with *MALAT1* in both CD4+ and CD8+ T cells as networks (Fig. 3A). Upon clustering these using *k*-means (*k* = 3), the resulting clusters showed FDR corrected enrichment for “Cell Division”, “Oxidative phosphorylation,” and “Response to Cytokine” (Fig. 3A).

Next, we investigated the gene lists using gene set enrichment analysis [53] using the hallmark gene sets to look at signatures within our gene lists. *MALAT1* anti-correlated genes were significantly enriched for cell-cycle targets of E2F transcription factors, genes regulated by MYC, progression through cell division (G2M) for CD4+ and CD8+ T-cells suggesting the *MALAT1* anti-correlated signature might play a role in proliferation and cell cycle progression (CD4 and CD8, Fig. 3B). Further, these gene sets showed enrichment for hypoxia, oxidative phosphorylation, and glycolysis for both CD4+ and CD8+ T cell whereas genes for DNA repair were only enriched in *MALAT1* anti-correlated gene list for CD4+ T cells (CD4, Fig. 3B).

Genes upregulated in response to IFN-γ and IFN-α signaling were hallmarks uniquely associated with CD4+ T cells (unique to CD4, Fig. 3B). Genes involved in complement were associated with both CD4+ and CD8+ T cells while genes associated with xenobiotic metabolism were associated with CD4+ T cells (unique to CD4, Fig. 3B). Further, *MALAT1* anti-correlating genes uniquely in CD8+ T cells were enriched for genes involved in the p53 pathway and those regulated in response to TNF via NF-κB (unique to CD8, Fig. 3B).

We looked at the top 20 genes uniquely anti-correlated to *MALAT1* in CD4+ T cells for STRINGDB interactions and found that genes related to response to TNF/IL-1 such as PSMA5, NFKBIA, and Ubiquitin cross-reactive protein (ISG15) (Fig. 3C). On the other hand, in CD8+ T cells, MALAT1 uniquely anti-correlates with genes associated with membrane targeting of proteins along with genes involved in CD8+ T-cell exhaustion like GNLY, GZMB, and HAVCR2 (Fig.3D).

### MALAT1 and MKI67 anti-correlate in COVID-19 post-mortem lung tissue

**Figure 4.**
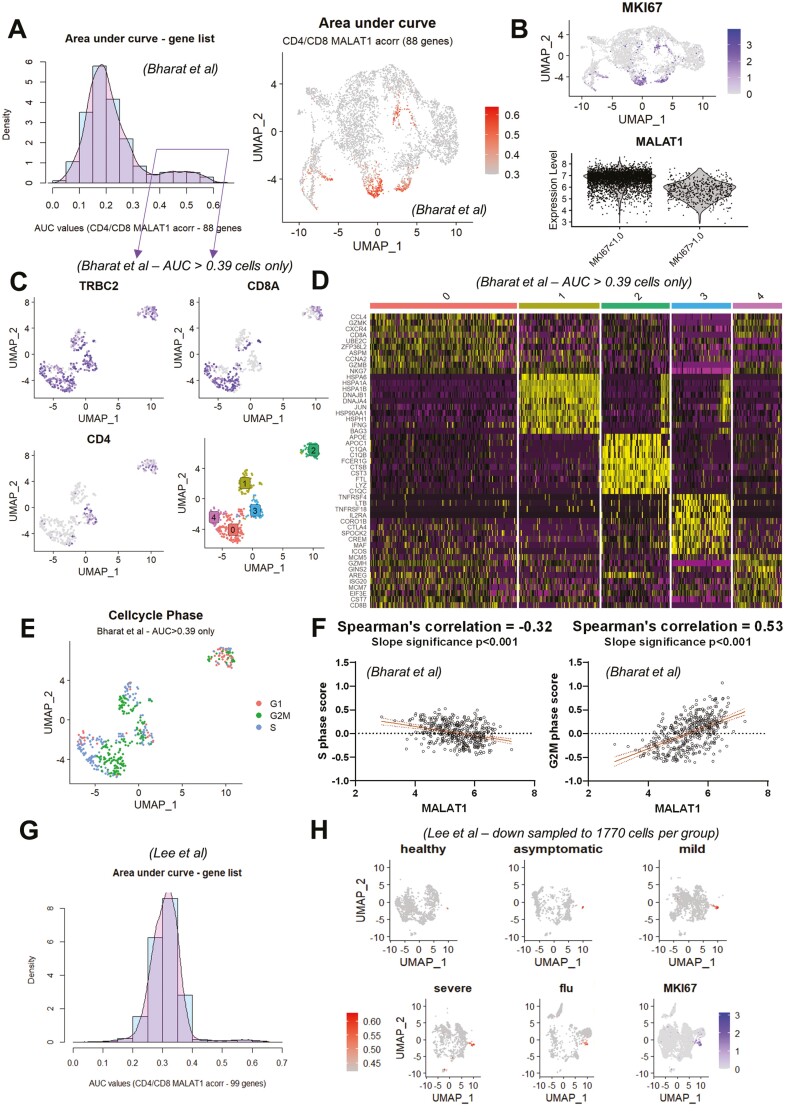
*MALAT1* expression is reduced in proliferating T cells from lung digests. (**A**) Histogram showing ‘area under recovery curve’ score for *MALAT1* anti-correlated gene list (number of genes = 88) for single cells. UMAP plot highlighting cells with AUC score from B that are greater than 0.39. (**B**) UMAP plot depicting MKI67 and a violin plot showing *MALAT1* expression in groups created based on *MKI67* levels of greater or less than 1.0. (**C**) UMAP plot depicting TRBC2, CD8A, and CD4 gene expression along with cluster identities of proliferative T cells (subset based on AUC score greater than 0.39 from A). (**D**)Heatmap showing top 10 genes expressed in each imputed cluster in (C). (**E**) UMAP plot depicting imputed cell cycle phase for each T cell. (**F**) Scatter plots and linear regression between imputed S-phase score (left) and G2/M score (right) per cell and *MALAT1*. *P*-values indicates the significance of the slope of the regression. (**G**) Histogram showing ‘area under recovery curve’ score for *MALAT1* anti-correlated gene list (number of genes = 99) for PBMCs. (**H**) UMAP plot highlighting cells with AUC score in PBMCs from healthy volunteers and asymptomatic, mild, and severe COVID-19 patients and individuals with flu. The last UMAP shows *MKI67* levels per cell.

The above cell-type gene signature and pathway analyses indicated a potential link between *MALAT1* expression and T-cell proliferation. To further test this, we checked if the *MALAT1* anti-correlated gene list signature common to CD4+ and CD8+ T cells (Fig. 3A) identified in BAL samples was sufficient to mark proliferating T cells in lung tissue. For this purpose, we analyzed a COVID-19 explant/post-mortem lung scRNA seq dataset [49]. We pre-filtered barcodes labeled as “T cells” from the dataset and used the top 100 common genes that are anti-correlated with *MALAT1* (Fig. 3A), of which 88 genes were found in Bharat *et al*., to calculate the ‘area under recovery curve’ or AUC [54] for each cell to calculate enriched gene set activity per cell (histogram, Fig. 4A) to identify gene list enrichment. Thresholding the AUC score (at AUC >= 0.39) based on the bimodality in AUC distribution (histogram, Fig. 4A), the cells were highlighted on a UMAP plot (Fig. 4A). The high AUC score highlights proliferating T cells as indicated by their corresponding *MKI67* expression in UMAP space and lower *MALAT1* levels is associated with cells with high *MKI67* levels (Fig. 4B).

Interestingly, the proliferative T cells in post-mortem lung tissue appeared diverse in terms of their position in UMAP space. To investigate this further, we examined a subset of these cells (AUC scores >= 0.39) and visualized canonical T-cell markers in two-dimensional UMAP space (Fig. 4C). We further re-clustered these cells (Fig. 4C, bottom right) to understand whether the heterogeneity in proliferative T cells with a high AUC score (Fig. 4A) translates in terms of differential gene expression (Fig. 4D).

We found that that cluster 0 (Fig. 4C) comprised of CCL4, CXCR4 expressing CD8 + T_EM_ (CD8A, GZMK, GZM, MKG7, and GZMH) cells (Fig. 4D, Supplementary Table S1). Cluster 1 was comprised of IFNG+ γδT cells (Fig. 4D) expressing TRDC and GNLY (Supplement Table S1). Cluster 2 markers appeared to have a strong macrophage-like gene signature with complement genes, FCER1G, and LYZ being upregulated in this cluster (Fig. 4D). As this cluster also expressed T-cell markers (Fig. 4C), we postulated that these were doublets and were not further analyzed. Cluster 3 was enriched in markers for CD4 T_REG_ such as IL12RA and CTLA4 (Fig. 4C) whereas cluster 4 comprised of CD8+ (CD8B, Fig. 4C) cells with targets of E2F transcription factors such as MCM5.

We then tested whether *MALAT1* expression levels were consistently lower in proliferative cells and whether this was dependent on their cell cycle state. We calculated a score based on genes involved in cell cycle progression[55] including those involved in the S, G2/M, and G1 phases. In Fig. 4E, we show how these proliferative T-cell clusters (Fig. 4C, bottom right) comprise cells in S, G2/M, and G1 phases. We then calculated Spearman’s correlation between the imputed cell phase score and *MALAT1* and found that *MALAT1* levels are anti-correlated with the imputed S phase score and strongly positively correlated with the G2M score (Fig. 4F).

To test if our findings were limited to tissue T cells, we examined a COVID-19 PBMC dataset [50], using the above-defined top 100 genes that anti-correlate with *MALAT1* we identified a small proportion of cells within this dataset that expressed these genes differentially (99/100 genes were found in the dataset, Fig. 4G). In fact, this signature also picks out PBMCs from influenza patients, suggesting that this is a hallmark feature of T-cells responding to infection. As in the case of lung T-cells, these AUC > 0.42 cells (Fig. 4G and H) are found to be neighborly in UMAP space and express MKI67 (Fig. 4H).

**Figure 5.**
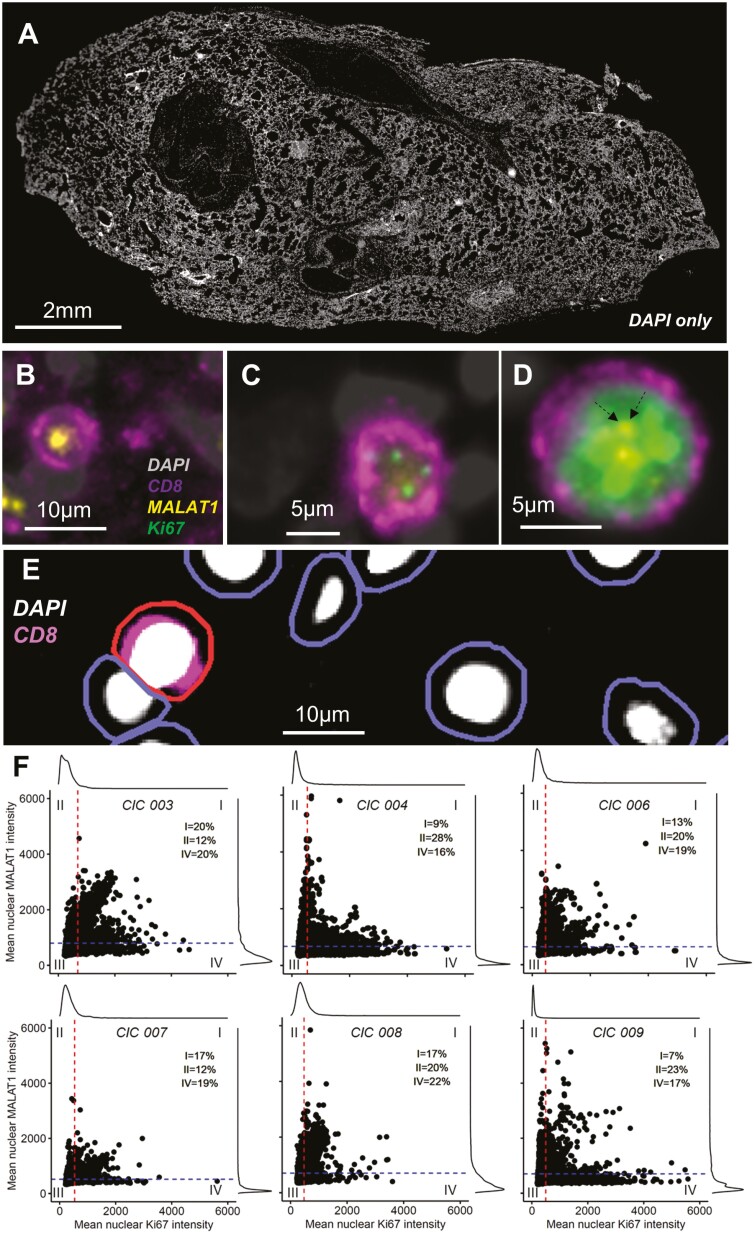
*MALAT1* expression is reduced in proliferating CD8+ T cells post-mortem tissue. (**A**) DAPI stained (grey) whole lung autopsy section. (**B–D**), Immunofluorescence and RNAScope images showing nucleus in grey (DAPI), MKI67 protein in green, CD8 surface protein in purple and *MALAT1* RNA in yellow. Images show representative cells negative for MKI67 and positive for *MALAT1* (**B**), weakly positive for MIK67 and with undetectable *MALAT1* (**C**) and highly expressing MKI67 along with *MALAT1* (**D**). Dotted arrows in (D) highlight *MALAT1* positive signal for clarity. (**E**) Strategy employed to detect cells wherein first nucleus was identified and then cell boundaries using QuPath (see Methods) and a positive cell was detected (shown here in red) based on CD8 fluorescence intensity of the cell. (**F**) Scatter/histogram plots per autopsy along with horizontal and vertical lines drawn at the mean nuclear *MALAT1* (793, 671, 629, 516, and 703) and MKI67 (685, 530, 457, 534, and 476) intensities, respectively. The two straight lines divide the plot into four quadrants, into regions that include cells that highly express MKI67 only (IV), *MALAT1* only (II), both (I), or neither (III).

To test the above findings *in situ*, we examined post-mortem lung sections from the UK Coronavirus Immunology Consortium (UK-CIC) (patient_meta_data, Supplementary Table S1 and Milross *et al*., in prep). We analyzed lung autopsy sections (*n* = 6) and representative sections stained with DAPI are shown in Fig. 5A. We concentrated on CD8+ T cells due to their roles in COVID-19 pathology [16] and the fact that *MALAT1* expression co-varied with both proliferation and exhaustion markers in these cells (Fig. 3D). We determined CD8 expression and MKI67 (as a marker for non-senescent cells that may be in any of G1, S, G2, and M phases) by immunofluorescence along with *MALAT1* by RNAScope. We found, qualitatively, that MALAT1 was seldom co-expressed with MKI67 unless MKI67 levels were high (Fig. 5B–D). Interestingly, this suggested some correlation between MALAT1 and MKI67 when the latter was more highly expressed. This may be related to the *MALAT1* expression correlation we observed with the G2M phase T-cell score (Fig. 4F). We next performed quantitative analysis using QuPath (Fig. 5E). For all tested samples we observed distinct MKI67-hi/MALAT1-lo populations, with the majority of highest MKI67 expressing CD8+ T cells (mean nuclear intensity > 2000) showing low *MALAT1* levels. We also found double-positive CD8+ cells that co-express *MALAT1* and MKI67 (Fig. 5F). These double-positive cells may be explained by the particular phase of the cell, as it has been shown that MKI67 is not a binary marker for proliferation but a graded marker for proliferation/senescence [56]. In general, however, we found that when MALAT1 expression is high then MKI67 expression is low and vice versa (Fig. 5F) across all tested samples.

## Discussion

*MALAT1* is one of the most abundant non-ribosomal RNA transcripts in mammalian transcriptomes. Despite an increasing understanding of how *MALAT1* upregulation contributes to cancer development and progression [57], less is known about its physiological functions in non-transformed cells. Recent work from our and other laboratories has indicated that MALAT1 plays a role in T-cell function and that, in preclinical models, antigenic activation of naïve T-cells results in suppression of *MALAT1* expression [42–46]. Here, we used published annotated transcriptomic datasets in COVID-19 to specifically look at T-cell phenotypes ranging from naïve to effector memory and exhausted and found that *MALAT1* is negatively correlated with a core gene signature in T cells, which in turn is linked to cellular proliferation. Using post-mortem lung autopsy samples, we experimentally validated this association and showed that MKI67+ proliferative CD8+ cells are characterized by low *MALAT1* expression.

T-cell proliferation can be spontaneous or homeostatic[58] and the conditions that regulate the same vary between CD4+ and CD8+ T cells [59]. CD8+ T-cell proliferation is essential with rapid proliferation in response to interaction with a foreign peptide but also during homeostasis if T-cell numbers fall below a threshold [60]. The former, however, progresses through to a CD8 effector memory phenotype [61]. In fact, it has been demonstrated in CD8+ T cells, that a T-central memory phenotype is marked by a higher number of prior divisions than the effector memory T-cell pool [62–64]. The replicative history of T cells is closely connected to its functional repertoire[64]. Interestingly, CD8+ T exhausted cells in COVID-19 are connected via the CD8 T_N_, CD8+ T_EM_ lineage (using pseudo time analysis) and have higher levels of proliferation markers [48]. Indeed, using Wauters Mol, *et al*., 2021 dataset, we note that CD8 + T_EX_ have a corresponding lower *MALAT1* level with increased expression of *MALAT1* anti-correlating genes (Fig. 2C).

*MALAT1* has been long associated with enhanced proliferation in cancer [35, 65] and the lack of the gene is shown in human diploid lung fibroblasts to have a reduction in their proliferation with an arrest at the G1/S phase with an increase in genes involved in the p53 pathway [41]. Interestingly, in T cells we observe a physiological downregulation of *MALAT1* that anticorrelates with the S-phase score of cells (Fig. 4F), suggesting *MALAT1* suppression may be a consequence of T-cell proliferation. Interestingly, overall *MALAT1* levels anti-correlate to HALLMARK_P53_PATHWAY (Fig. 3B) and is unique to CD8 + T cells. While *MALAT1* in this work has been shown to anti-correlate with a cell’s S-phase score (Fig. 4F), it has been shown that many lincRNAs peak during the S phase in human epithelial cells leading to transcriptional regulation during cell cycle progression [66]. In that study, it was found that *MALAT1* peaks close to the beginning of G2/M [66]. In this respect, we found *MALAT1* levels to correlate with G2M score in T cells (Fig. 4F), which indicates the similarity of T cells to epithelial cells in terms of *MALAT1* expression during the cell cycle.

We find that more genes anti-correlate with *MALAT1* than those that correlate (Fig. 2A). Whether this is due to the direct effects of *MALAT1* through its roles in gene regulation [67] will need to be further tested. However, it suggests that physiological regulation of *MALAT1* levels may alter gene expression of T cells that are known for their plasticity [68]. Further still, as cell proliferation is central to T-cell activation [69, 70], it will be interesting to investigate how a lack of *MALAT1* during proliferation may shape T-cell function upon subsequent activation and differentiation. We have previously reported that a lack of *MALAT1* results in lower levels of MAF and IL10 in mice and as a consequence, greater host resistance to infection or increased immunopathology [46]. Others have reported impaired CD8+ T-cell function upon MALAT1 loss [45]. Interestingly, MALAT1 mediates its function through interactions with proteins and potentially RNA, interactions which based on the results presented here would be expected to be altered in MALAT1-lo proliferating T cells. Genes that may be associated with shaping T-cell function post proliferation may indeed lie amid the *MALAT1* anti-correlated signature that we find in CD4+ and CD8+ T cells, especially those involved in cytokine response and oxidative phosphorylation (Fig. 3A). As an example we find HAVCR2 (TIM-3) which is a marker for T-cell exhaustion[71] anti-correlates with *MALAT1* (unique to CD8+ T cells, Fig. 3D). How these genes may vary between T-cell subsets such as CD8+ T-central memory where lowly divided cells are capable of mounting a better effector response upon re-infection[64] and exhausted CD8+ T-cell population with increased cell cycle markers like MKI67[3, 48] requires further investigation.

Taken together our results reveal that suppression of *MALAT1* expression is a feature of proliferating activated T cells. This means that *MALAT1*-associated functions are likely to be suppressed in proliferating T cells, but not necessarily that MALAT1 suppression drives the proliferation. There is a long list of reports supporting that MALAT1 promotes cell proliferation at least within the context of cancer cells[72]. Based on this, we speculate that one possibility is that MALAT1 downregulation following T-cell activation can be a potential mechanism to limit uncontrolled T-cell proliferation. This however will need to be experimentally confirmed in future studies. Mechanistically, MALAT1 might affect T-cell activation, proliferation, or differentiation through its role in post-transcriptional regulation, for example through direct interaction with several RNA-binding proteins[73], many of which are involved in T-cell proliferation and differentiation[74, 75]. The *MALAT1*-linked gene signatures identified here provide an initial insight into the potential functional consequences of *MALAT1* suppression in human T cells, forming the foundation for further mechanistic studies on the function of this highly expressed lincRNA in T cells within and beyond viral infection.

## Methods

### Datasets

Single-cell RNA seq data from healthy and COVID-19 patients from gene expression omnibus accession number GSE145926 which is referred to throughout the paper as Liao *et al*. [47] and T-cell barcodes (using metadata from the original publication) were subset and used further for analysis. Dataset Wauters *et al*. [48] was obtained from https://lambrechtslab.sites.vib.be/en/data-access. Specifically, the file T_NKT_cells.counts.rds was downloaded to use as counts matrix. Single-cell data for barcodes with ‘COVID19’ as metadata were included in the downstream analysis from the Wauters *et al*. dataset.

Finally, accession number GSE158127 [49] was used to analyze post-mortem T cells from the lungs and for validation. Further, GSE149689 [50] was used to look at *MALAT1* signatures in PBMCs and in flu.

Cell cycle genes were not regressed prior to dimensionality reduction and downstream analysis in any of the datasets to show proliferating T cells as a separate cluster owing to their distinct cell cycle-related gene expression.

### *In silico* T-cell quality check and phenotype identification

Single T-cell transcriptomes from Liao *et al*. and Wauters *et al*. were loaded as Seurat (v4.0.5) objects and the latter Seurat object’s metadata describing T-cell phenotypes were used to impute T-cell phenotypes in Liao *et al*. using the functions FindTransferAnchors() and Transferdata(). Next, single transcriptomes with greater than 5% mitochondrial genes were discarded from downstream analysis. Next, counts from both Seurat objects were regressed using percentage of mitochondrial genes, ribosomal genes, total RNA count, and number of unique features using method “glmGamPoi” which is available as an R package with the same name (https://bioconductor.org/packages/release/bioc/html/glmGamPoi.html). Finally, the anchors between the two Seurat Objects were found (functions SelectIntegrationFeatures() and FindIntegrationAnchors()) to then integrate (IntegrateData()) them into a single integrated Seurat object.

### Dimensionality reduction

Principal components analysis was performed on the integrated Seurat object (3000 variable features). Top 30 PCA components were used to cluster the data by a K-nearest neighbor clustering using FindClusters() with a resolution parameter of 0.8. UMAP was performed on the PCA space and single cells were represented on UMAP axes and colored by their cluster membership.

### Correlation analysis

The correlation of all genes with *MALAT1* was calculated using cor.test() from the stats package in R (4.1.1) implemented with Spearman’s ranked correlation method. The level of significance associated with a correlation was set at 0.05. Correlation values between −0.1 and 0.1 (both included) were excluded.

### Network and gene set enrichment analysis

Network analysis of gene lists was performed on String-DBGene set enrichment analysis was performed on STRING (https://string-db.org/). Gene set enrichment analysis (GSEA, http://www.gsea-msigdb.org/gsea/msigdb/annotate.jsp) using the option to ‘Investigate Gene Sets’ to search for significant (*P-value corrected*) overlaps with Hallmark gene sets, GO biological process, cellular component, and molecular function.

### Area under curve

Gene list enrichment in cells was calculated using the R package AUCell, originally published as a part of SCENIC [54]. Expression matrices as obtained from the Seurat object were provided to the function AUCell_buildRankings() to build cell rankings which were then used to calculate an ‘area-under-recovery-curve’ for the provided gene list. AUC score thresholds were selected based on visual inspection and are indicated in the relevant figure.

### RNAScope and immunofluorescence

Post-mortem autopsy sections from UK-CIC first wave cohort (CIC003-9) were obtained on glass slides and stained for CD8, MKI67, and DAPI. *MALAT1* was probed on the same section using RNAScope (Bio-techne) FISH assay as per the manufacturer’s instructions.

### QuPath

All images were acquired on a Zeiss AxioScan.Z1 slide scanner. Exposure times and threshold settings for all three channels were used for each of the images. Images in the CZI format were loaded on QuPath-0.3.2 [76]. Whole images were analyzed for co-expression of MKI67, CD8, and *MALAT1* at single-cell resolution, and count data were analyzed. CD8 + cells were detected using the module ‘positive cell detection’ using DAPI as a counterstain to draw nuclei and cell boundaries. Cellular CD8 intensity was then used to detect positive cell types. Data was exported and then further investigated in R. Cells with a circularity score of less than 0.75 were excluded and expression positivity for MKI67 and MALAT1 was determined by selecting only those cells that had a maximum pixel intensity greater than the minimum detected intensity.

## Supplementary Material

uxad034_suppl_Supplementary_Figures

uxad034_suppl_Supplementary_Tables

## Data Availability

All single-cell RNA seq data used in this study are publicly available and their source is described in methods under the sub-section “Datasets”. The code used to analyze data is available here—https://github.com/jipsi/malat_proliferation/ and all processed/unprocessed Rds files used in this study are available at—https://doi.org/10.5281/zenodo.7506637. Images and analyses generated by this study can be made available upon request.

## Abbreviations

AUC: area under recovery curve
BAL: broncheoalveolar lavage
CD: cluster of differentiation
CD4/CD8_EM: CD4/CD8 memory cells
CD8_EMRA: CD8 recently activated memory cells
CD8_EX: CD8 exhausted
CD8_RM: CD8 resident memory cells
COVID-19: coronavirus disease
GSEA: gene set enrichment analysis
LincRNA: long intergenic RNA
lncRNA: long non-coding RNA
MALAT1: metastasis-associated lung adenocarcinoma transcript 1
PBMCs: peripheral blood mononuclear cells
Regulatory T cells: Treg
SARS-CoV-2: severe acute respiratory syndrome coronavirus 2
scRNA: single-cell RNA sequencing
TCRs: T-cell receptors;
Th1: T helper type 1
UK-CIC: UK coronavirus immunology consortium;
UMAP: uniform manifold approximation and projection.
