# Supplementary material for: Downregulation of MALAT1 is a hallmark of tissue and peripheral proliferative T cells in COVID-19
Source: Clin Exp Immunol. 2023 Mar 3;212(3):262–75. doi: 10.1093/cei/uxad034 (PMC10243916; doi:10.1093/cei/uxad034)
Supplement: uxad034_suppl_Supplementary_Figures [file uxad034_suppl_supplementary_figures.docx]

**SUPPLEMENTARY TO:**

**Down-regulation of the *MALAT1* is a hallmark of tissue and peripheral proliferative T cells in COVID-19**

Shoumit Dey^1*^, Helen Ashwin^1^, Luke Milross^2^, Bethany Hunter^3^, Joaquim Majo^4^, Andrew J Filby^3^, Andrew J Fisher^2,5^, Paul M. Kaye^1^, Dimitris Lagos^1*^

^1^Hull York Medical School and York Biomedical Research Institute, University of York, UK.

^2^Newcastle University Translational and Clinical Research Unit, Faculty of Medical Sciences, Newcastle University, UK.

^3^Flow Cytometry Core Facility and Innovation, Methodology and Application Research Theme, Newcastle University Biosciences Institute, Newcastle University, UK.

^4^Department of Cellular Pathology, Newcastle Upon Tyne Hospitals NHS Foundation Trust, Newcastle upon Tyne, UK

^5^ Institute of Transplantation, Newcastle upon Tyne Hospitals NHS Foundation Trust

Newcastle upon Tyne, UK.

*Correspondence: [Dimitris.lagos@york.ac.uk](mailto:Dimitris.lagos@york.ac.uk), or [shoumit.dey@york.ac.uk](mailto:shoumit.dey@york.ac.uk), Hull York Medical School, University of York, York, YO105DD,

Short running title: Low MALAT1 is a hallmark proliferative T cells in COVID-19

Keywords: T cell, proliferation, MALAT1, COVID-19, lncRNA

**
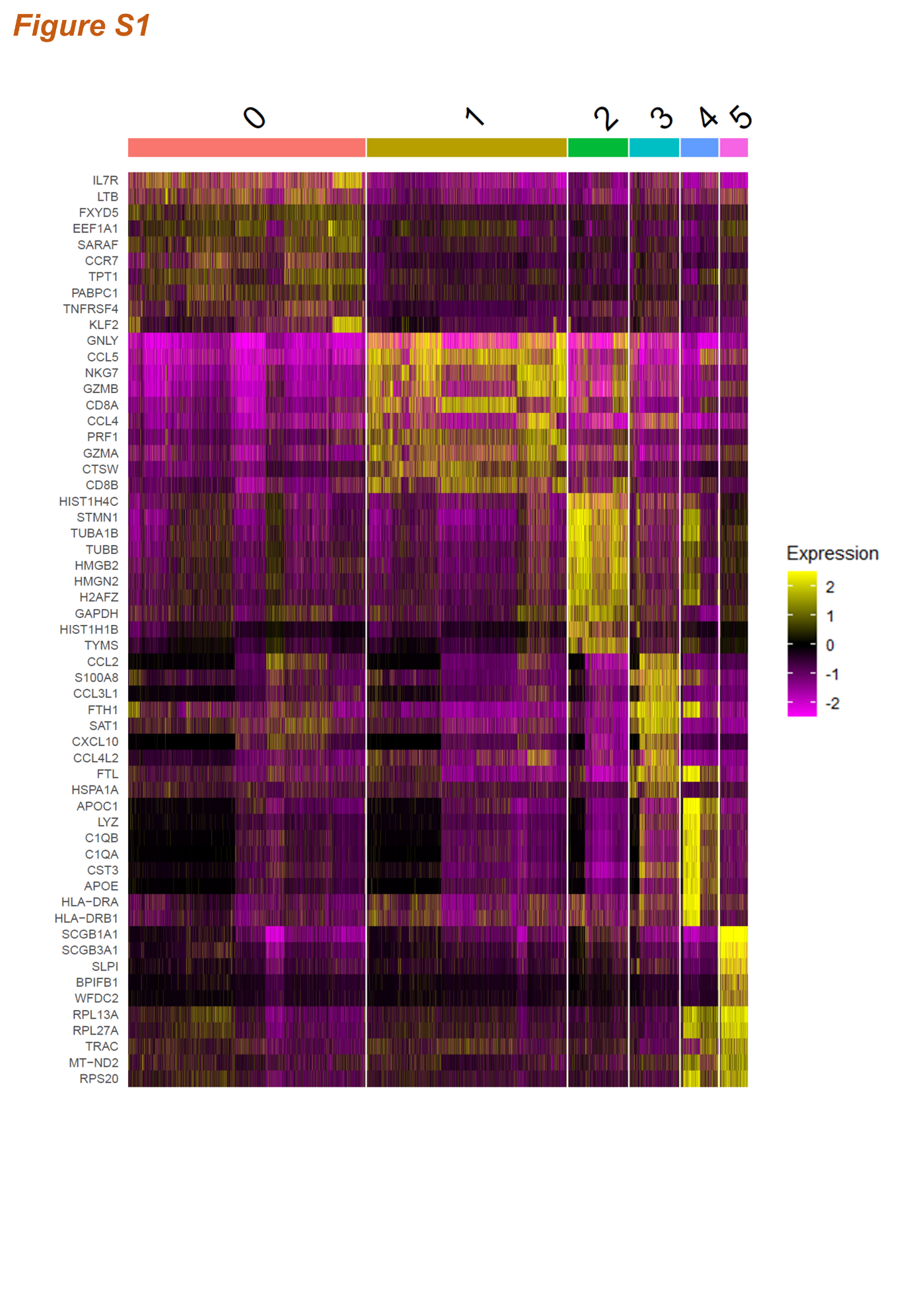
**

**Figure S1: Gene expression across imputed cell clusters**

Heatmap showing top 10 genes expressed in each cluster as identified in the integrated dataset.

**
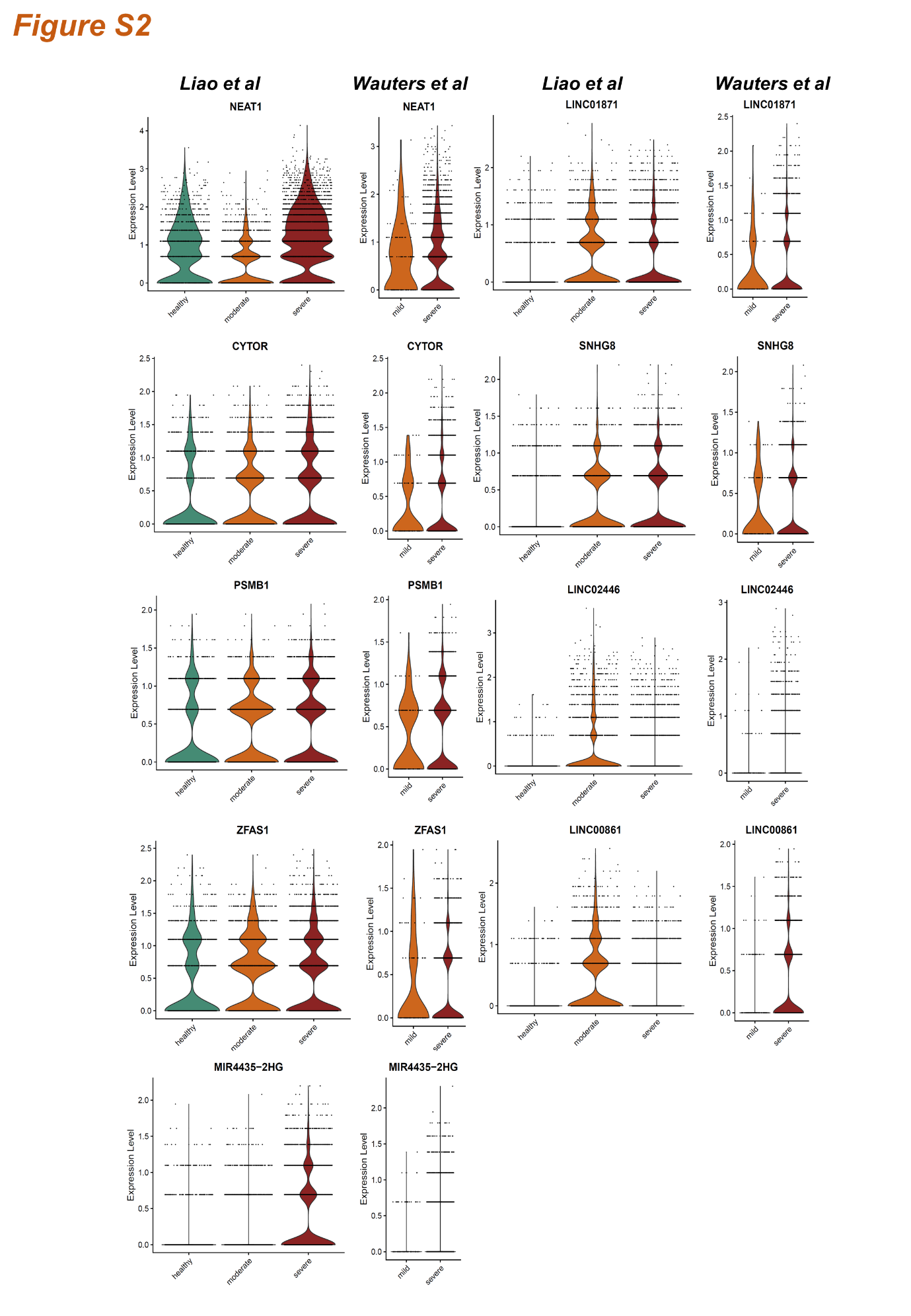
**

**Figure S2: LncRNA expression across disease severity and dataset**

Violin plots indicating gene expression per cell for the top 2-10 (excluding *MALAT1*) highly expressed lncRNAs in the integrated dataset.
